# Temporal Variation in Air Pollution Concentrations and Preterm Birth—A Population Based Epidemiological Study

**DOI:** 10.3390/ijerph9010272

**Published:** 2012-01-18

**Authors:** David Olsson, Magnus Ekström, Bertil Forsberg

**Affiliations:** 1 Division of Occupational and Environmental Medicine, Department of Public Health and Clinical Medicine, Umeå University, 901 87 Umeå, Sweden; Email: bertil.forsberg@envmed.umu.se; 2 Centre of Biostochastics, Swedish University of Agricultural Sciences, 901 83 Umeå, Sweden; Email: magnus.ekstrom@slu.se

**Keywords:** pregnancy, preterm birth, vehicle emissions, ozone, nitrogen dioxide

## Abstract

There is growing evidence of adverse birth outcomes due to exposure to air pollution during gestation. However, recent negative studies are also reported. The aim of this study was to assess the effect of ozone and vehicle exhaust exposure (NO_2_) on the length of the gestational period and risk of preterm delivery. We used data from the Swedish Medical Birth Registry on all vaginally delivered singleton births in the Greater Stockholm area who were conceived during 1987–1995 (n = 115,588). Daily average levels of NO_2_ (from three measuring stations) and ozone (two stations) were used to estimate trimester and last week of gestation average exposures. Linear regression models were used to assess the association between the two air pollutants and three exposure windows, while logistic regression models were used when analyzing associations with preterm delivery (<37 weeks gestation). Five percent were born preterm. The median gestational period was 40 weeks. Higher levels of ozone during the first trimester were associated with shorter gestation as well as with an elevated risk of preterm delivery, the odds ratio from the most complex model was 1.06 (95% CI: 1.00–1.13) per 10 μg/m^3^ increase in the mean daily 8-h maximum concentration. Higher levels of ozone during the second trimester were associated with shorter gestation but the elevated risk of preterm delivery was not statistically significant. Higher levels of ozone and NO_2_ during the last week of gestation were associated with a shorter duration of gestation and NO_2_ also with preterm delivery. There were no significant associations between first and second trimester NO_2_ exposure estimates and studied outcomes. The effect of first trimester ozone exposure, known to cause oxidative stress, was smallest among women who conceived during autumn when vitamin D status, important for fetal health, in Scandinavian women is the highest.

## 1. Background

There is an ever growing body of evidence that ambient air pollution levels during gestation have adverse effects on birth outcomes [1,2,3,4,5,6,7,8,9,10,11,12]. Maternal exposure to carbon monoxide (CO), or motor vehicle exhaust, late in the pregnancy was associated with preterm birth in Vancouver, Canada and in California [4,5,6]. Late pregnancy exposure to fine and respirable particulate matter (PM_2.5_, PM_10_) has also been associated with an increased risk of preterm delivery in California [5,6]. Early pregnancy exposure to ozone (O_3_) and PM_10_ increased the risk of preterm delivery in Brisbane, Australia [13]. Increased concentration of several pollutants during the first and third trimester were associated with preterm birth in the Republic of Korea [9]. A study from the Czech Republic, where sulphur dioxide levels (SO_2_) were rather high, found SO_2_ to be associated with preterm birth regardless of period of exposure [12]. Additionally, exposure to particles during early gestation as well as exposure to NO_x_ during early or late gestation was associated with preterm birth. It has been suggested that there may be some seasonality in the effects from exposure to air pollution during different exposure windows of gestation on the duration of gestation [10].

However, there is no unambiguous answer as to which specific elements of pollution or during which period of pregnancy, has an impact on pregnancy outcomes. Sram *et al.* conclude that the evidence is strong enough to infer causality of air pollution on low birth weight, LBW (birth weight < 2.5 kg), while the evidence of association between air pollution and preterm birth is deemed insufficient to infer causality [2]. More recently, Bosetti *et al.* reviewed a variety of studies and still found that the studies on maternal exposure to respirable particles during pregnancy do not provide conclusive evidence of an association with the risk of preterm birth and low birth weight [14]. Other authors conclude that for traffic related pollution, the association with an increased risk of preterm birth seems to be consistent [7]. However, several recent studies were negative: in the Dutch PIAMA birth cohort there was no significant association between preterm birth and NO_2_ during first trimester, last month or entire pregnancy [15]. Neither was there any significant association between NO_2_ and preterm birth in a study from California [3]. From Sydney an unexplained protective effect of NO_2_ has been reported [10] and in Valencia the dose-response relationship was V-shaped [16]. The few studies of O_3_ have not found robust associations [3,4,10,13]. Preterm birth is a risk factor not only in early life, but is also associated with respiratory and cardiovascular disease later in life [17,18]. Thus more and large studies are needed, especially from Europe where most of the reported studies of air pollution exposure have been small.

Studies of maternal air pollution exposure and preterm birth/LBW build on different types of exposure variables; concentrations at monitoring stations, modelled concentrations or proximity to sources (roads). The contrasts in exposure variables may be completely spatial or completely temporal or both, but more spatial than temporal [7,8,10]. 

The aim of this study was to assess the association between exposure to O_3_ and duration of gestational and preterm delivery as well as the association with vehicle exhaust, using NO_2_ as indicator, during different windows of gestation in the Greater Stockholm area. 

## 2. Population and Methods

All vaginally delivered singleton births by mothers in the Greater Stockholm area who conceived during 1988–1995 were included in the study (n = 115,588). The number of inhabitants in this area was approximately 1.6 million and the area was approximately 3,500 km^2^, with approximately 40% of the population residing in the City of Stockholm, an area of approximately 200 km^2^. The information was extracted from the Swedish Medical Birth Registry and included date of birth, birth weight, length at birth, smoking habits of the mother, parity, gestational age and sex of the child. Birth date and information on gestational age were used to determine date of conception and the period for the first (gestational week 1–13) and second (gestational week 14–26) of each trimester of pregnancy. Since the preterm born have a shorter third trimester, we chose not to calculate exposure for this period of varying duration. We instead calculated exposure during the last week at risk of preterm birth. Gestational age was calculated using information on date of last menstruation and from ultrasound examinations.

The air pollution data was provided by the City of Stockholm Environment and Health Administration. We used citywide daily average levels to calculate trimester averages and averages over the last week of pregnancy for NO_2_ (daily 1-hour maxima) and O_3_ (daily 8-hour maxima). O_3_ was measured at one urban background station (Södermalm above roof) and the regional background station (Norr Malma), while NO_2_ was measured at three different urban background stations (above roof) within a 1.5 km radius (Södermalm, Hornsgatan, Sveavägen). The daily citywide average was calculated from measured concentrations, or when one station was missing, after imputing values from a linear regression against the other stations as for the APHEA-2 Study [19]. 

Logistic regression was used to examine if the pollutants influenced the risk for preterm birth (<37 weeks gestation). Both crude models and adjusted models were fitted. In order to improve the quality of the models, they were adjusted for: smoking habits (non-smoker, moderate smoker (1–9 cigarettes/day), heavy smoker (10+ cigarettes/day) and unknown smoking habits), parity (0, 1, 2, 3 or more), and sex. We adjusted for time-varying variables; temperature (°C) and relative humidity (%), as they may confound the study of pollution effects. Additionally we adjusted for seasonal trend by fitting a cyclic regression spline on day of year for conception. Finally, we included a long term trend by fitting a cubic regression spline for conception year. We examined the effects of exposure to O_3_ and NO_2_ during the first and second trimesters and during the last week at risk of preterm birth. The exposure effects for the different time frames were modelled both individually and simultaneously. We also studied interaction-effects between air pollution and season of conception in the multi-pollutant fully adjusted models. The seasons were defined as; spring (March to May), summer (June to August), autumn (September to November) and winter (December to February).

The effect of the pollution exposures on duration of gestation (as a continuous outcome, in weeks) was assessed using multiple linear regression models, where adjustment was made for the same set of potential confounders mentioned above. The different exposure windows we studied were; the first and second trimesters of pregnancy, and the last week of pregnancy.

We estimated 95% confidence intervals (CI) for all association parameters (slopes and odds ratios). All analyses were made using R statistical software package (2.13.0) [20]. 

## 3. Results

**Figure 1:**
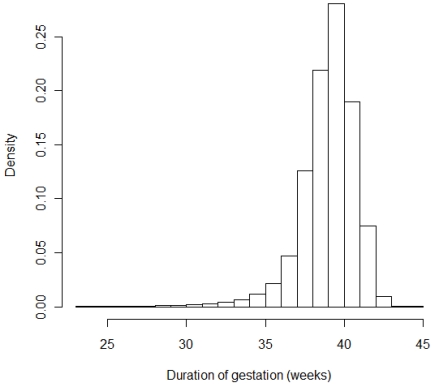
Histogram of duration of gestation.

**Figure 2:**
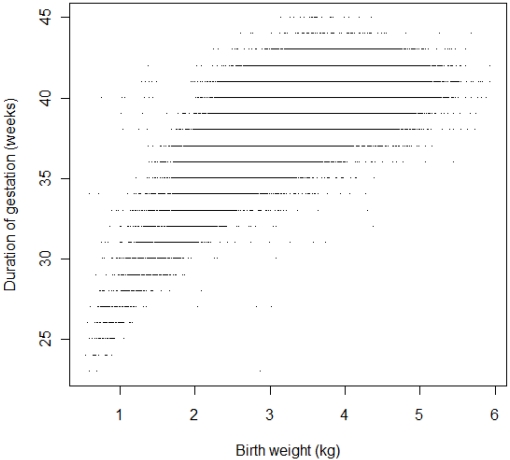
Duration of gestation plotted against birth weight.

About one half of the mothers were primiparous and about a third gave birth to their second child. 5.3% of the births were preterm. The distribution of duration of gestation was skewed towards the lower end of the spectrum (Figure 1). The variability in birth weight was greater among gestations with longer durations (Figure 2). 

The average pollution levels and meteorology were relatively similar for different time frames of pregnancy (Table 1). There were moderate negative correlations between O_3_ and NO_2_ (Table 2), and positive correlations between O_3_ and ambient temperature. O_3_ and relative humidity were strongly negatively correlated. NO_2_ and ambient temperature had a moderate negative correlation. NO_2_ and relative humidity was positively correlated. Both O_3_ and NO_2_ had a slight positive correlation between first and second trimester averages. The correlation between the first trimester and the last week of gestation averages was negative for both pollutants. The correlation between the second trimester O_3_ and the last week of gestation O_3_ averages was negative. There was no correlation between the second trimester NO_2_ and the last week of gestation NO_2_ averages. 

**Table 1:** Descriptive statistics of air pollutants and meteorological variables.

*Time frame*	*Pollutant*	*Mean ^a^*	*SD*	*Median*	*IQR ^b^*
First trimester	O_3_	57.1	13.3	55.3	20.4
Second trimester	O_3_	56.7	13.7	54.7	20.9
Last week ^c^	O_3_	59.0	17.5	58.4	25.3
Last week ^d^	O_3_	58.2	17.2	57.5	25.1
First trimester	NO_2_	38.5	5.4	38.3	6.0
Second trimester	NO_2_	38.9	5.4	38.6	5.8
Last week ^c^	NO_2_	38.3	8.6	37.6	11.0
Last week ^d^	NO_2_	38.2	8.5	37.7	10.9
First trimester	Temperature	7.9	6.3	7.4	12.2
Second trimester	Temperature	7.3	6.2	6.3	11.4
Last week ^c^	Temperature	7.7	7.2	7.1	12.4
Last week ^d^	Temperature	7.4	7.2	6.4	11.9
First trimester	Humidity	69.9	9.3	70.9	15.2
Second trimester	Humidity	70.5	9.1	71.8	14.7
Last week ^c^	Humidity	70.0	11.9	71.1	18.2
Last week ^d^	Humidity	69.9	11.7	71.1	18.0

^a^ Units are µg/m^3^ for the air pollutants, °C for temperature and % for relative humidity. ^b^ IQR is equal to the difference between the 75th and 25th percentiles. ^c^ Last week of gestation. ^d^ Last week at risk of preterm birth.

**Table 2:** Correlation between O_3_, NO_2_ and meteorological averages.

	First trimester O_3_	Second trimester O_3_	Last week ^a^ O_3_	Last week ^b^ O_3_	First trimester NO_2_	Second trimester NO_2_	Last week ^a^ NO_2_	Last week ^b^ NO_2_
Second trimester O_3_	0.13							
Last week ^a^ O_3_	−0.34	−0.45						
Last week ^b^ O_3_	−0.50	−0.32	0.71					
First trimester NO_2_	−0.43	0.16	−0.03	0.11				
Second trimester NO_2_	−0.33	−0.39	0.30	0.30	0.25			
Last week ^a^ NO_2_	0.29	0.11	−0.26	−0.34	−0.09	0.03		
Last week ^b^ NO_2_	0.29	0.00	−0.18	−0.26	−0.07	0.03	0.48	
First trimester temperature	0.62	−0.61	0.06	−0.18	−0.53	0.09	0.20	0.28
Second trimester temperature	0.69	0.56	−0.73	−0.77	−0.10	−0.50	0.37	0.33
Last week ^a^ temperature	−0.79	−0.08	0.54	0.61	0.44	0.36	−0.40	−0.39
Last week ^b^ temperature	−0.78	0.17	0.27	0.54	0.49	0.27	−0.32	−0.40
First trimester relative humidity	−0.82	0.24	0.26	0.46	0.42	0.19	−0.20	−0.25
Second trimester relative humidity	−0.30	−0.79	0.69	0.61	−0.11	0.40	−0.28	−0.19
Last week ^a^ relative humidity	0.55	0.41	−0.71	−0.62	−0.30	−0.36	0.22	0.20
Last week ^b^ relative humidity	0.65	0.23	−0.49	−0.70	−0.41	−0.30	0.28	0.23

^a ^Last week of gestation. ^b^ Last week at risk of preterm birth.

The proportion of preterm births was higher among smoking mothers (Table 3). The proportion of preterm births was higher in the subset of women who did not have any information on smoking than in the other subsets. The proportion of preterm births was higher among primiparous women than for women giving birth for the second time. Among women giving birth to their third or subsequent child the proportion of preterm births was higher than among primiparous women or women giving birth to their second child. There was only a minor difference between boys and girls in the proportion of preterm birth. 

There was no clear seasonality in how preterm births were distributed over the year, there was however a slightly elevated proportion of preterm births among the mothers who conceived during Spring or Autumn (Table 3). The proportion of preterm births was somewhat lower during 1989 and 1990, the two years with the lowest first trimester O_3_ averages, compared to the other years, and the proportion of preterm births was slightly elevated in 1994, when first trimester O_3_ levels were the highest in this study.

**Table 3:** Proportion preterm delivery within levels of covariates in the adjusted analysis.

Variable	N	Proportion of total population, %	Proportion Preterm Delivery, %	Duration of gestation, weeks (sd)	Average first trimester O_3_ level, µg/m^3^(sd)	Average first trimester NO_2_ level µg/m^3^(sd)
*Smoking habits*						
Non smoker	87,213	75.5	4.84	39.5 (1.8)	57.2 (13.3)	38.3 (5.3)
Moderate smoker	12,251	10.6	5.36	39.5 (1.9)	56.9 (13.4)	39.0 (5.5)
Heavy smoker	7,520	6.5	6.76	39.3 (2.0)	57.2 (13.4)	39.0 (5.6)
Unknown smoking habits	8,601	7.4	8.96	39.1 (2.3)	56.6 (13.2)	39.3 (5.7)
*Parity*						
First child	55,295	47.8	5.33	39.5 (1.9)	57.0 (13.3)	38.6 (5.4)
2nd child	39,919	34.5	4.24	39.5 (1.7)	57.2 (13.3)	38.4 (5.4)
3rd child	14,524	12.6	6.27	39.3 (1.9)	57.0 (13.3)	38.5 (5.4)
4th or subsequent child	5,852	5.2	10.34	38.9 (2.2)	57.1 (13.3)	38.3 (5.3)
*Sex*						
Male	59,192	51.2	5.44	39.5 (1.9)	57.1 (13.3)	38.5 (5.4)
Female	56,396	48.8	5.20	39.4 (1.8)	57.1 (13.3)	38.5 (5.4)
*Season of conception*						
Spring	28,505	24.7	5.43	39.4 (1.9)	72.8 (6.7)	36.2 (4.5)
Summer	31,819	27.5	5.21	39.5 (1.9)	58.2 (9.3)	36.2 (5.2)
Autumn	28,876	25.0	5.42	39.4 (1.9)	42.8 (6.1)	41.4 (5.7)
Winter	26,388	22.8	5.25	39.5 (1.8)	54.4 (9.1)	40.7 (3.5)
*Year of conception*						
1988	14,165	12.3	5.52	39.5 (1.8)	61.8 (13.1)	43.9 (6.0)
1989	14,478	12.5	4.91	39.5 (1.8)	50.1 (13.2)	43.6 (5.2)
1990	14,719	12.7	5.08	39.5 (1.8)	52.4 (12.6)	36.1 (5.3)
1991	15,071	13.0	5.27	39.4 (1.9)	54.7 (9.8)	37.9 (3.1)
1992	15,065	13.0	5.42	39.5 (1.9)	57.1 (14.7)	37.8 (2.9)
1993	14,640	12.7	5.46	39.4 (1.9)	56.1 (9.7)	37.5 (2.8)
1994	14,100	12.2	5.56	39.4 (1.9)	65.1 (12.9)	36.2 (3.5)
1995	13,350	11.5	5.39	39.4 (1.9)	60.1 (13.0)	35.0 (5.3)
Total	115,588		5.32			

There was a clear association between O_3_ levels during the first trimester and preterm delivery, both in the unadjusted and adjusted models (Table 4). The association was stronger in the multiple pollutant models than in the crude model. The Odds Ratio, OR, was equal to 1.08 per a 10 µg/m^3^ increase in O_3_ in the individual exposure window model, CI = (1.03, 1.13). In the multiple exposure window model the OR was 1.06 per 10 µg/m^3^ increase in O_3_, CI = (1.00, 1.13), and the crude OR was 1.03 per 10 µg/m^3^ increase in O_3_, CI = (1.01, 1.05). Higher second trimester O_3_ was positively associated with preterm birth, although the 95% confidence intervals included the null in all models. We did not observe any clear association between elevated O_3_ levels towards the end of gestation and preterm birth. First and second trimester NO_2_ levels were non-significant with OR < 1. Higher levels of NO_2_ during the last week at risk of preterm birth was positively associated with preterm birth in all models.

**Table 4:** Crude and adjusted ORs for preterm delivery per 10 µg/m^3^ increase in pollutant concentration.

Time frame	Pollutant	Crude OR (95% CI)	ORs and (95% CI) from multiple pollutant model ^a^. Exposure windows studied individually	ORs and (95% CI) from multiple pollutant model ^a^. Exposure windows studied simultaneously
First trimester	O_3_	1.03 (1.01, 1.05)	1.08 (1.03, 1.13)	1.06 (1.00, 1.13)
Second trimester	O_3_	1.01 (0.99, 1.03)	1.02 (0.97, 1.07)	1.05 (0.98,1.12)
Last week	O_3_	0.99 (0.98, 1.01)	1.01 (0.98, 1.04)	1.02 (0.98, 1.05)
First trimester	NO_2_	0.96 (0.92, 1.01)	0.95 (0.88, 1.03)	0.95 (0.87, 1.04)
Second trimester	NO_2_	0.95 (0.91, 1.00)	0.96 (0.88, 1.04)	0.93 (0.85, 1.02)
Last week	NO_2_	1.05 (1.02, 1.08)	1.07 (1.03, 1.11)	1.06 (1.02, 1.11)

^a^ Adjusted for maternal smoking, parity, sex of the child, temperature, relative humidity, seasonal variation and long term trend.

**Table 5:** Crude and adjusted slope estimates for duration of gestation (in weeks) per 10 µg/m^3^ increase in pollutant concentration.

Time frame	Pollutant	Crude slope estimate (95% CI)	Slope estimates and (95% CI) from multiple pollutant model ^a^. Exposure windows studied individually	Slope estimates and (95% CI) from multiple pollutant model ^a^. Exposure windows studied simultaneously
First trimester	O_3_	−0.02 (−0.03, −0.01)	−0.06 (−0.08, −0.04)	−0.05 (−0.08, −0.02)
Second trimester	O_3_	−0.01 (−0.02, −0.01)	−0.03 (−0.05, −0.01)	−0.07 (−0.10, −0.04)
Last week	O_3_	0.01 (0.00, 0.01)	−0.02 (−0.03, −0.01)	−0.02 (−0.03, −0.01)
First trimester	NO_2_	0.04 (0.02, 0.06)	0.04 (0.00, 0.07)	0.01 (−0.03, 0.05)
Second trimester	NO_2_	0.05 (0.03, 0.07)	0.02 (−0.01, 0.06)	0.04 (−0.01, 0.08)
Last week	NO_2_	−0.03, (−0.04, −0.02)	−0.04 (−0.05, −0.02)	−0.04 (−0.06, −0.02)

^a^ Adjusted for maternal smoking, parity, sex of the child, temperature, relative humidity, seasonal variation and long term trend.

The associations were very similar in the analyses of gestational period. In the fully adjusted model high O_3_ levels were associated with a shorter duration of gestation, regardless of window of exposure (Table 5). The strongest association, in the multiple pollutant—multiple exposure windows model, was observed for second trimester O_3_ and duration of gestation. A 10 µg/m^3^ increase in O_3_ was associated with a 0.07 weeks reduction of the duration of gestation, CI = (−0.10, −0.04). Higher first and second trimester NO_2_ levels had a tendency to be positively associated with gestation, but the 95% confidence intervals for the slope estimates excluded the null only for the crude models. Exposure to high levels of O_3_ and NO_2_ during the last week of gestation was associated with a shorter duration of gestation.

The observed association between first trimester O_3 _and preterm birth was modified by season, the ORs for preterm delivery ranged from 1.07, CI = (0.98, 1.16), during autumn to 1.13, CI = (1.05, 1.21), during spring per 10 µg/m^3^ increase in O_3_ during the first trimester (Table 6). The weakest association for first trimester exposure to O_3_ was for those pregnancies where the conception day happened during autumn, and this was the only season-specific O_3_ effect where the confidence interval included the null. 

**Table 6:** Season-specific ORs for preterm delivery and β-coefficients for gestational period (in weeks) per 10 µg/m^3^ increase in 1st trimester O_3_ concentration.

Season	Crude OR (95% CI)	Adjusted OR (95% CI) ^a^	Crude slope estimates (95% CI)	Adjusted slope estimates (95% CI) ^a^
Spring	1.10 (1.02, 1.18)	1.13 (1.05, 1.21)	−0.06 (−0.09, −0.03)	−0.07 (−0.10, −0.04)
Summer	1.05 (0.99, 1.10)	1.13 (1.02, 1.26)	−0.03 (−0.06, 0.00)	−0.04 (−0.07, −0.02)
Autumn	1.05 (0.96, 1.14)	1.07 (0.98, 1.16)	−0.06 (−0.09, −0.02)	−0.06 (−0.09, −0.03)
Winter	1.15 (1.08, 1.21)	1.12 (1.04, 1.20)	−0.07 (−0.10, −0.04)	−0.06 (−0.09, −0.03)

^a^ Multiple pollutant model adjusted for maternal smoking, parity, sex of the child, first trimester temperature, first trimester relative humidity, first trimester NO_2_, a seasonal spline and long term cubic regression spline. High exposure to O_3_ during the first trimester was associated with a shorter duration of gestation, regardless of season of conception, although the association was weakest during summer (Table 5).

## 4. Discussion

We found an association between first trimester O_3_ exposure and increased risk of preterm birth, which was consistent with descriptive data for the different years. We have studied the effects of temporal fluctuations in air pollution exposure between pregnancies. This approach gives us the ability to detect effects better for exposures that vary more strongly in time than in space. That is, this study is well suited to detect effects of O_3_, but not so well suited to detect the full effects from air pollutants with an additional within city variation, *i.e.*, NO_2_. At this latitude, O_3_ levels are mainly dependent on the incoming air masses, and the local formation is very limited [21]. At the same time, the regional background concentrations of pollutants such as NO_2_ are low, and the fluctuations in concentrations are highly dependent on the dispersion of emissions from local traffic in the city. This means that periods of stagnation will increase exhaust levels everywhere in the city, though more in some streets and districts than in others. As such, there will have been some misclassification of NO_2_ exposures, which as non-differential typically would tend to produce attention in slopes and odds ratios. We use a few stations only for a quite large area. However, air pollution data from the same monitoring stations have for this population shown significant associations in time-series studies of daily mortality and hospitalisations in APHEA-2 and other studies, indicating their validity [22]. 

The Medical Birth Registry has existed in Sweden since 1973, is almost complete (99%) and of very high quality [23]. Because of this it is very unlikely that missing data has had any impact on the results of this study. One quality issue we observed with the Medical Birth Registry was the lack of information on smoking habits. In 7.4% of the births in this study we did not have information on smoking status. Possible explanations to these missing records are; unwillingness to respond to this question during the first antenatal care visit, failure to report information from antenatal care by the birthing centre or antenatal care started in a later stage of pregnancy. This is probably more common among women with social problems. There is of course the issue of migration. Exposure misclassification will occur for women who moved to the study area during gestation. However, although such movements may bias associations towards the null, we do not believe these migration patterns could confound the associations driven by studying temporal fluctuations in air pollution concentrations. The only criterion for a child to be entered into the study was to be a vaginally delivered singleton birth during the study period; this means that we have no restriction as to how many times one woman is represented. A consequence of this is that the observations are not independent, and there may be some individual factors that strongly predicts duration of gestation, for example some genetic predisposition. However, the direction and magnitude of the associations did not change when restricting the models to primiparous women only (Appendix A).

A typical problem in air pollution studies is potential confounding, and in studies of birth outcome socioeconomic status, lifestyle, country of origin *etc*. may differ between areas with more or less air pollution. As we only study the influence of temporal variability in the air pollutants, we primarily need to worry about potential confounding for variables that vary in time in a similar way [11,24]. We did not observe any confounding effect on the results for NO_2_. We observed some confounding from the meteorological factors and time of conception on the results for first trimester O_3_, inflating the ORs from 1.06 per IQR increase in the unadjusted model to 1.16 per IQR increase in the adjusted model. Season of conception, temperature and relative humidity could be good proxies for sunlight exposure, which is correlated with ground level O_3_ formation. Sunlight is associated with maternal vitamin D status. Maternal vitamin D levels are important for fetal health, and vitamin D deficiency during pregnancy has been associated with preeclampsia [25]. Inflammation in early pregnancy is associated with an increased risk of preterm delivery [26]. Vitamin D deficiency is associated with increased circulating inflammatory proteins, and vitamin D status seems to modulate C-reactive protein levels [27]. In a recent study exposures to PM and O_3_, but not NO_2_, were associated with increased C-reactive protein concentrations in early pregnancy [28]. Vitamin D status has a clear seasonal variation in Scandinavian women, and is best in the autumn [29]. In this study the effect of first trimester O_3_ levels was smallest among women who conceived during the autumn. 

One of the limitations in this study is the lack of adjustment for socioeconomic factors. There are no good proxies for socioeconomic status in the Medical Birth Registry. Socioeconomic status varies more strongly in space than in time, at least in a shorter time span. It might be plausible that there is confounding from temporal variations in socioeconomic status on the associations between air pollution and preterm delivery. Most of this confounding should be accounted for by adjusting for time, as both the variation of air pollution and the variation of socioeconomic status are functions of time. Although the risk of confounding from socioeconomic status is limited, the overall performance of the models would likely improve by adjusting for socioeconomic status.

Our finding of an early pregnancy effect of O_3_ is similar, albeit smaller, than the findings of Hansen *et al.* [13]. Liu *et al.* found an OR of 1.08 for a 10 ppb increase in first month O_3_ in their unadjusted model, but this effect was reversed, OR = 0.98 per 10 ppb increase, when adjusting for possible confounders; maternal age, parity, infant sex, birth weight and season of birth [4]. The first finding is similar to the first trimester crude effect in this study, but the results diverge in the adjusted models. The difference in results may be due to difference in how seasons were defined, Liu *et al.* adjusted for season of birth (which may be modified by preterm birth) while we adjusted for season of conception. No associations between early gestation exposure to O_3_ were found in the two studies from the southern California [3,5]. The inconsistent findings could be caused by differences in susceptibility (e.g., different anti-oxidant status in different populations), differences in exposure patterns (e.g., high prevalence of air conditioning reduces O_3_ exposure) and differences in the pollution mix (e.g., negative correlation with primary combustion components).

There could be two explanations to the overall null effect of early and mid-gestation NO_2_ concentrations. One explanation is that there is no effect; the other is that we fail to catch important variability in exhaust exposure during the first and second trimester as we were unable to take into account spatial variation in exposure. However, in the Californian studies there was spatial resolution in the exposure data, still they found no or weak indications of an adverse effect of early gestation exposure to higher ambient NO_2_ levels [3,5]. The observation of an adverse association of exposure to higher NO_2_ levels during the last period of gestation has been observed in several studies [3,4,5,9,13]. There have also been observations of a null effect of exposure to higher NO_2_ levels during the last part of pregnancy [10]. The correlations between NO_2_ and other pollutants, such as particles, may differ between the different study locations. 

All season-specific estimates of exposure to higher O_3_ levels during the first trimester were associated with an elevated risk of preterm delivery and a shorter duration of gestation, although the association with preterm birth was slightly weaker for conceptions in autumn than for conceptions during any other season. This strengthens our belief that there seems to be a negative effect of early gestation exposure to O_3_ on the duration of the gestational period. The season-specific findings are not in accordance with the findings of Jalaludin *et al.*, who found statistically significant positive associations with O_3_ levels during the first trimester for women who conceived during spring or summer [10]. The association with O_3_ levels we found is robust in that we observe the effect regardless of whether we study duration of gestation or preterm delivery. 

The pathway by which we believe O_3_ may cause preterm delivery is through oxidative stress. Oxidative stress caused by exposure to air pollution may lead to inflammatory response in the respiratory system [30]. Expression of proinflammatory cytokines could disrupt trophoblast invasion during the formation of the placenta [13,31]. This could lead to preeclampsia, which is an important predictor of preterm birth. Alternatively systemic oxidative stress may affect the embryo at the earliest phase of development, which may cause intra-uterine growth retardation or preterm birth [32]. Exposure to air pollution in a later stage of gestation may cause disturbances in cord blood flow or increased susceptibility for maternal infections, which may cause premature rupture of membranes leading to an earlier birth [5]. 

We have shown that although the air pollution level is moderate in Sweden there is an association between exposure to air pollution during pregnancy and reduction of gestational age. Our results support the observation that there is some seasonality in the association with air pollution. Further studies are needed to find out more about these associations and possible mechanisms. 

How important are these effects of moderate air pollution from a public health point of view? A comparison with smoking shows that the impact of air pollution must be taken seriously. 6.5% of the mothers (7,520) were heavy smokers with a prevalence of preterm birth of 6.76% compared to 4.84% preterm among non-smoking mothers. This corresponds to 144 preterm births attributed to heavy smoking. The estimated attributable fraction preterm births associated with heavy smoking was 2.1%. 10.6% of the mothers were moderate smokers with a prevalence of preterm birth of 5.36%, and 64 preterm births attributed to moderate smoking. The adjusted ORs (heavy smoking 1.37, moderate smoking 1.12) would give similar numbers. The upper quartile of first trimester O_3_ exposure has a mean value of 27.7 μg/m^3^ over the lowest quartile, which corresponds to an OR of 1.18. The fraction of preterm births attributed to the upper quartile of first trimester O_3_ exposure was 7.8% (corresponding to 129 preterm births) for this exposure category. The upper quartile of last week NO_2_ exposure has a mean value of 16.9 µg/m^3^ over the lowest quartile, which corresponds to an OR of 1.11. The estimated fraction of preterm births attributable to the highest quartile of late pregnancy NO_2_ exposure was 5.2% (corresponding to 85 preterm births) for this exposure category.

Heavy smokers had a reduction in duration of gestation estimated to approximately 0.19 [95% CI = (−0.23, −0.14)] weeks, while moderate smokers had no reduction. The upper quartile of first trimester O_3 _exposure has a mean value of 27.7 μg/m^3^ over the lowest quartile, which corresponds to 0.13 [95% CI = (−0.21, −0.05)] weeks shorter gestation (using the adjusted slope). However, this is a mean value, but the real effect is likely not evenly distributed. The average reduction in duration of gestation for women exposed to levels within the highest quartile of last week NO_2_ compared to the lowest quartile was 0.068 [95% CI = (−0.096, −0.040)] weeks. 

## 5. Conclusions

The results found in this study indicate that there is an early (first trimester) effect of O_3_ in accordance with suggested pathways for the early exposure window. The effect of O_3_ was smallest among women who conceived during the autumn when the vitamin D levels in Scandinavian women should be the highest. We observed an adverse effect of exposure to elevated NO_2_ levels in late gestation on preterm delivery. However, no spatial variation in NO_2_ was modelled. There remains a need to study these associations further with subject specific exposure assessments. However, the total size of these effects supports improvements in air quality also in a moderately polluted region. 

## Supplementary Files

Supplementary File 1: PDF-Document (PDF, 158 KB)
