# Supplementary material for: Temporal Variation in Air Pollution Concentrations and Preterm Birth—A Population Based Epidemiological Study
Source: Int J Environ Res Public Health. 2012 Jan 18;9(1):272–85. doi: 10.3390/ijerph9010272 (PMC3315074; doi:10.3390/ijerph9010272)
Supplement: Supplementary File 1: — PDF-Document (PDF, 158 KB) [file ijerph-09-00272-s001.pdf]

Int. J. Environ. Res. Public Health **2012**, *9*, 272-285; doi:10.3390/ijerph9010272

OPEN ACCESS

International Journal of
Environmental Research and Public Health
ISSN 1660-4601
www.mdpi.com/journal/ijerph

Supplementary Information

Temporal Variation in Air Pollution Concentrations and Preterm Birth—A Population Based Epidemiological Study

David Olsson [1,*], Magnus Ekström [2] and Bertil Forsberg [1]

[1] Division of Occupational and Environmental Medicine, Department of Public Health and Clinical Medicine, Umeå University, 901 87 Umeå, Sweden; E-Mail: bertil.forsberg@envmed.umu.se

[2] Centre of Biostochastics, Swedish University of Agricultural Sciences, 901 83 Umeå, Sweden; E-Mail: magnus.ekstrom@slu.se

* Author to whom correspondence should be addressed; E-Mail: david.olsson@envmed.umu.se; Tel.: +46-90-785-3479; Fax: +46-90-786-9995.

Received: 16 December 2011; in revised form: 9 January 2012 / Accepted: 13 January 2012 / Published: 18 January 2012

Abstract: There is growing evidence of adverse birth outcomes due to exposure to air pollution during gestation. However, recent negative studies are also reported. The aim of this study was to assess the effect of ozone and vehicle exhaust exposure (NO_2) on the length of the gestational period and risk of preterm delivery. We used data from the Swedish Medical Birth Registry on all vaginally delivered singleton births in the Greater Stockholm area who were conceived during 1987–1995, n = 115,588. Daily average levels of NO_2 (from three measuring stations) and ozone (two stations) were used to estimate trimester and last week of gestation average exposures. Linear regression models were used to assess the association between the two air pollutants and three exposure windows, while logistic regression models were used when analyzing associations with preterm delivery (<37 weeks gestation). Five percent were born preterm. The median gestational period was 40 weeks. Higher levels of ozone during the first trimester were associated with shorter gestation as well as with an elevated risk of preterm delivery, the odds ratio from the most complex model was 1.06 (95% CI 1.00–1.13) per 10 μg/m^3 increase in the mean daily 8-h maximum concentration. Higher levels of ozone during the second trimester were associated with shorter gestation but the elevated risk of preterm delivery was not statistically significant. Higher levels of ozone and NO_2 during the last week of gestation were associated with a shorter duration of gestation and NO_2 also with preterm delivery. There were no significant associations between first and second trimester NO_2 exposure estimates and studied outcomes. The effect of first trimester ozone exposure, known to

cause oxidative stress, was smallest among women who conceived during autumn when vitamin D status, important for fetal health, in Scandinavian women is the highest.

Keywords: pregnancy; preterm birth; vehicle emissions; ozone; nitrogen dioxide

Table S1. Crude and adjusted ORs for preterm delivery per 10 μg/m^3 increase in pollutant concentration for primiparous women.

Time frame	Pollutant	Crude OR (95% CI)	ORs and (95% CI) from multiple pollutant model [a]. Exposure windows studied individually	ORs and (95% CI) from multiple pollutant model [a]. Exposure windows studied simultaneously
First trimester	O_3	1.04 (1.01, 1.07)	1.09 (1.02, 1.15)	1.06 (0.98, 1.15)
Second trimester	O_3	1.00 (0.98, 1.03)	1.00 (0.94, 1.06)	1.06 (0.96,1.16)
Last week	O_3	0.99 (0.96, 1.01)	1.01 (0.98, 1.04)	1.01 (0.97, 1.06)
First trimester	NO_2	1.01 (0.95, 1.08)	1.01 (0.91, 1.13)	1.01 (0.90, 1.13)
Second trimester	NO_2	0.99 (0.92, 1.06)	0.94 (0.84, 1.05)	0.91 (0.81, 1.02)
Last week	NO_2	1.07 (1.03, 1.12)	1.04 (0.99, 1.09)	1.06 (1.00, 1.12)

[a] Adjusted for maternal smoking, parity, sex of the child, temperature, relative humidity, seasonal variation and long term trend.

Table S2. Crude and adjusted slope estimates for duration of gestation (in weeks) per 10 μg/m^3 increase in pollutant concentration for primiparous women.

Time frame	Pollutant	Crude slope estimate (95% CI)	Slope estimates and (95% CI) from multiple pollutant model [a]. Exposure windows studied individually	Slope estimates and (95% CI) from multiple pollutant model [a]. Exposure windows studied simultaneously
First trimester	O_3	−0.02 (−0.03, −0.01)	−0.06 (−0.09, −0.03)	−0.03 (−0.07, 0.01)
Second trimester	O_3	−0.01 (−0.02, 0.00)	−0.04 (−0.07, −0.01)	−0.08 (−0.12, −0.03)
Last week	O_3	0.01 (0.00, 0.02)	−0.02 (−0.04, 0.00)	−0.02 (−0.04, 0.00)
First trimester	NO_2	0.02 (−0.01, 0.04)	0.03 (−0.02, 0.08)	0.02 (−0.04, 0.07)
Second trimester	NO_2	0.02 (0.00, 0.05)	0.02 (−0.03, 0.07)	0.05 (−0.01, 0.10)
Last week	NO_2	−0.03, (−0.05, −0.01)	−0.02 (−0.05, 0.00)	−0.03 (−0.05, 0.00)

[a] Adjusted for maternal smoking, parity, sex of the child, temperature, relative humidity, seasonal variation and long term trend.

© 2012 by the authors; licensee MDPI, Basel, Switzerland. This article is an open access article distributed under the terms and conditions of the Creative Commons Attribution license (http://creativecommons.org/licenses/by/3.0/).
